# Diagnosis features of pediatric Gaucher disease patients in the era of enzymatic therapy, a national-base study from the Spanish Registry of Gaucher Disease

**DOI:** 10.1186/s13023-017-0627-z

**Published:** 2017-05-03

**Authors:** Marcio Andrade-Campos, Pilar Alfonso, Pilar Irun, Judith Armstrong, Carmen Calvo, Jaime Dalmau, Maria-Rosario Domingo, Jose-Luis Barbera, Horacio Cano, Maria-Angeles Fernandez-Galán, Rafael Franco, Inmaculada Gracia, Miguel Gracia-Antequera, Angela Ibañez, Francisco Lendinez, Marcos Madruga, Elena Martin-Hernández, Maria del Mar O’Callaghan, Alberto Pérez del Soto, Yolanda Ruiz del Prado, Ignacio Sancho-Val, Pablo Sanjurjo, Miguel Pocovi, Pilar Giraldo

**Affiliations:** 1 Haematology Department, Miguel Servet University Hospital, Zaragoza, Spain; 2 CIBER de Enfermedades Raras (CIBERER), Instituto Salud Carlos III, Zaragoza, Spain; 3 Traslational Research Unit, Aragon Institute of Health Research (IISAragon), Zaragoza, Spain; 4 Hospital Sant Joan de Deu, Barcelona, Spain; 5 Pediatric Department, San Jorge Hospital, Huesca, Spain; 6 Pediatric Department, La Fe University Hospital, Valencia, Spain; 7 Hospital Clinico Universitario Virgen de la Arrixaca, Murcia, Spain; 8 Pediatric Department, Manises Hospital, Valencia, Spain; 9 Haematology Department, Los Arcos del Mar Menor University Hospital, Murcia, Spain; 10 Haematology Department, Virgen del Puerto Plasencia, Plasencia, Spain; 11 Haematology Department, Punta Europa Hospital, Cádiz, Spain; 12 Pediatric Department, Miguel Servet University Hospital, Zaragoza, Spain; 13 Hospital Universitario Doctor Peset, Valencia, Spain; 14 Haematology Department, Complejo Hospitalario Albacete, Albacete, Spain; 15 Pediatric Department, Torrecárdenas Hospital, Almeria, Spain; 16 Neurology Department, Hospital Universitario Virgen del Rocio, Sevilla, Spain; 17 Pediatric Department, !2 Octubre University Hospital, Madrid, Spain; 18 Institut de Recerca Pediàtrica-Hospital Sant Joan de Déu (IRP-HSJD), CIBERER, Barcelona, Spain; 19 Haematology Department, Virgen del Rocío University Hospital, Sevilla, Spain; 20 Pediatric Department, Hospital San Millan y San Pedro, La Rioja, Spain; 21 Haematology Department, Alcañiz Hospital, Teruel, Spain; 22 Pediatric Department, Cruces University Hospital, Bilbao, Spain; 23 Biochemistry and Molecular and Cellular Biology Department, Zaragoza University, Zaragoza, Spain; 24 Spanish Foundation for the Study and Therapy of Gaucher Disease (FEETEG), Zaragoza, Spain; 25 Unidad de Investigacion Traslacional, Pta Baja, Hospital Universitario Miguel Servet, Paseo Isabel La Catolica 1-3, Zaragoza, 50009 Spain

**Keywords:** Children, Gaucher Disease, Enzymatic replacement therapy

## Abstract

**Background:**

The enzymatic replacement therapy (ERT) availability for Gaucher disease (GD) has changed the landscape of the disease, several countries have screening programs. These actions have promoted the early diagnosis and avoided many complications in pediatric patients. In Spain ERT has been available since 1993 and 386 patients have been included in the Spanish Registry of Gaucher Disease (SpRGD). The aim of this study is to analyze the impact of ERT on the characteristics at time of diagnosis and initial complications in pediatric Gaucher disease patients.

**Aim:**

To analyze the impact of ERT on the characteristics at time of diagnosis and initial complications in pediatric Gaucher disease patients.

**Methods:**

A review of data in SpRGD from patients’ diagnosed before 18 years old was performed. The cohort was split according the year of diagnosis (≤1994, cohort A; ≥1995, cohort B).

**Results:**

A total of 98 pediatric patients were included, GD1: 80, GD3: 18; mean age: 7.2 (0.17–16.5) years, 58 (59.2%) males and 40 (40.8%) females. Forty-five were diagnosed ≤ 1994 and 53 ≥ 1995. Genotype: N370S/N370S: 2 (2.0%), N370S/L444P: 27 (27.5%), N370S/other: 47 (48%), L444P/L444P: 7 (7.1%), L444P/D409H: 2 (2.0%), L444P/other: 3 (6.2%), other/other: 10 (10.2%). The mean age at diagnosis was earlier in patients diagnosed after 1995 (*p* < 0.001) and different between the subtypes, GD1: 8.2 (0.2–16.5) years and GD3: 2.8 (0.17–10.2) years (*p* < 0.001). There were more severe patients in the group diagnosed before 1994 (*p* = 0.045) carrying L444P (2), D409H (2), G377S (1), G195W (1) or the recombinant mutation. The patients’ diagnosed ≤1994 showed worse cytopenias, higher chance of bone vascular complications at diagnosis and previous spleen removal. The patients started ERT at a median time after diagnosis of 5.2 years [cohort A] and 1.6 years [cohort B] (*p* < 0.001).

**Conclusions:**

The early diagnosis of Gaucher disease in the era of ERT availability has permitted to reduce the incidence of severe and irreversible initial complication in pediatric patients, and this has permitted better development of these patients. This is the largest pediatric cohort from a national registry.

## Background

Gaucher Disease (GD)(OMIM#230800), the most common inherited lysosomal storage disorder is an autosomal recessively disease; the cause is mutations of the beta-acid-glucosidase gene located in chromosome 1 in the q21 region [1]. The gene alteration leads to a partial or total lack of glucocerebrosidase (GBA) enzymatic activity in the lysosome, GBA is a hydrolase responsible for the degradation of the glycosphingolipid complex glucosylceramide; the deficiency leads to an accumulation of complex molecules of glucosylceramide inside the lysosomes of reticuloendotelial cells, especially in viscera and bone [2–4].

Clinically, there are 3 sub-types according to neurological involvement. Type 1 GD non-neuronopathyc GD is the most common form of presentation in western countries, and as the name indicates there are no neurological manifestations, type 3 is the second most frequent and the clinical course is characterized by neurological manifestations like ataxia, saccadic eye movements, seizures and neurological impairment, and non-neurological features like heart valves infiltration, kyphosis and other characteristics described elsewhere [5, 6], type 2 GD or acute neuronopathic form is the most aggressive presentation of the GD, with severe neurological impairment early in life (newborns to 1 year old) with a short lifespan, usually around 2 years of age [6–8]. Pathophysiology studies reveal that these three types are a continuum of manifestations; from the more severe, the type 2 form, followed by the intermediate disease of type 3 to milder or non-symptomatic phenotypes of some type 1 patients [9, 10]. These multi-system manifestations are based on the grade of residual GBA enzymatic activity, and the association of some mutations with a high risk of neurologic-involvement [11, 12].

The time of diagnosis in GD is variable and directly related to the rate of substrate accumulation and severity presentation [13]. The clinical characteristics of pediatric GD patients are growth retardation, nasal hemorrhages, cytopenias, spleen enlargement and early bone crisis prior to the era of enzyme replacement therapy (ERT) the spleen was frequently removed to improve hematologic parameters, but had a negative impact on bone [14].

After more than two decades of ERT, few reports and guidelines have highlighted the efficacy of an optimal approach to early institution of ERT in pediatric GD patients. The large studies are supported by statistics extrapolating modelling-based studies from data from the International Collaborative Gaucher Group Registry (ICGGR) [15, 16] and several works from national cooperative groups that have summarize information from different cohorts [17–21]. However, the disease manifestations are impacted by the ethnicity/population characteristics and it is important to analyze what we have achieved in every-day clinical practice with current treatment and how the behavior of the disease has changed in the pediatric population, especially in children treated from the first years of life. Focusing on that, a country-based analysis from the Spanish Registry of Gaucher Disease had been performed and presented here.

## Methods

### Spanish registry

Since the establishment of the Spanish Registry of Gaucher Disease (SpRGD) coordinated by the “Fundacion Española para el Estudio y Terapeutica de la Enfermedad de Gaucher”, (FEETEG), a total of 386 GD patients have been reported in Spain (www.feeteg.org). All of them, or their parents in pediatric cases, had signed informed consent to be part of the registry and permit the use of their data. Approval from the ethics committee and institutional boards were obtained and all aspects are in accordance with the current version of the Helsinki Declaration; the management of the patients was the responsibility of the local physician in charge. From the FEETEG and GD unit, it is encouraged to follow the actual international and European Working Group on Gaucher Disease (EWGGD) recommendations guidelines [22–25] and to perform at least one visit to the GD unit for a general assessment.

### Inclusion criteria

A retrospective review of the available data from patients diagnosed in pediatric age (<18 years) and registered in the SpRGD-FEETEG was performed. All patients had a confirmed diagnosis of GD by determination of enzymatic activity B-acid glucosidase activity according to a protocol based on the artificial substrate incubation with 4 methylumbelliferyl-B-D-glucoside-sonicated extracts leukocyte [26], and identification of genetic mutations by sequencing of *GBA* gene [27]. All biological samples are kept in the Aragon Biobank and included in the Lysosomal Collection. The analyzed variables were: general demographic information, clinical manifestations, biomarkers, visceral & bone involvement, genotype, therapy and follow-up information. Period of study: May 1993 to April 2016.

### Study variables

#### Year of diagnosis

The cohort was split into 2 subgroups according to the diagnosis made before/in 1994 and after 1994. This decision was taken considering that in 1993, in the Miguel Servet University Hospital, the first adult patient started therapy in Spain. This milestone was followed by many efforts to improve the diagnosis and management of GD patients.

#### Hematologic values

Considering anemia, the hemoglobin levels (Hb) were defined according to international values (WHO) of normal for age and gender: birth to 6 months, <10.1 g/dL; 6 months to 2 years, <9.5 g/dL; 2 years to 12 years, <10.5 g/dL; more than 12 years, male, <12 g/dL and female, <11 g/dL.

For platelet counts, the normal value was considered above 120 × 10^9^ per L.

#### Visceral assessment

Liver and spleen volumes were assessed by physical examination and, when available, also for ultrasound and magnetic resonance. Considering the difficulty in summarizing data, the information was recorded as cm bellow low marginal costal or as multiples of normal (MN) size when a MRI was carried out.

#### Biomarkers studies

At diagnosis and for monitoring of patients chitotriosidase activity and the chemokine-C-motif ligand 18/Pulmonary-activation-regulated chemokine (CCL18/PARC) are part of our standard care. In addition we include the genotype of chitotriosidase to determine the presence of the duplication of 24 pair of bases in the 6th exon. The studies were done in plasma as previously described [28, 29].

Bone disease assessment: For bone disease evaluation, the history of chronic bone pain, bone crisis, bone lesions or deformities, and bone evaluations by X-ray or MRI prior to starting ERT were noted.

Bone crisis was defined according to the clinical criteria previously described [15], as “pain with acute onset that requires immobilization of the affected area and narcotics for the relief of pain and that may be accompanied by periosteal elevation, elevated white blood cell count, fever, or debilitation that lasts longer than 3 days.” Many were reported as suspected crisis and were registered according to the date the physician reported the event as occurring.

For bone mineral density, the results from the densitometry studies dual X-ray absorptiometry (DXA) or ultrasound quantification by broadband ultrasound attenuation (BUA) performed during routine follow-up were included.

#### Disease severity evaluation

For this purpose, the Pediatric Gaucher Severity Scoring system (PGS3) previously described by Kallisch S and Kaplan P. was applied [30].

#### Statistical analysis

All the data were collected into a database and analyzed using the SPSS v18.0 program. Because this is a national data registry, many patients were not initially assessed in our clinic, however from each patient, clinical and laboratory data were collected through the physician reports so there was no need to perform any modeling approach.

## Results

### Demographic characteristics

From the total of 386 patients diagnosed with Gaucher Disease in Spain, 124 (32.1%) were diagnosed bellow 18 years, 26 (20.9%) were type 2 and the other 98 (79.1%) suspected to have type 1 disease, of these, 18 (14.5%) had a genotype other than N370S, developed neurological symptoms and were re-classified as type 3. For this work we considered only GD1 and GD3 patients, (*n* = 98). There was male predominance (58, 59,2%). The mean age at diagnosis was 7.2 years (range. 0.2–16.5), about half of the patients (48, 46.9%) were diagnosed at 5 years or less. The majority of patients 86 (87.8%) had features of GD, and the diagnosis were established in the others (12, 12.9%) when family screening was performed.

A total of 45 (45.9%) patients were diagnosis during or before 1994 and 53 (54.1%) after 1994. Regarding GD3 patients 13/18 were diagnosed after 1994 Fig. 1. In the first 5 years of the ERT era 22 pediatric patients were diagnosed 4 had GD3. Table 1 summarizes more general characteristics and compares them with the time of diagnosis.

**Fig. 1:**
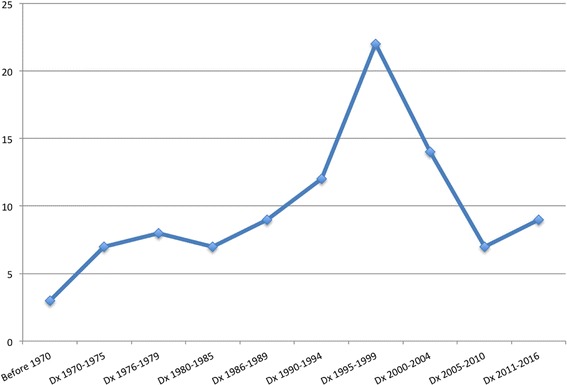
Timeline of pediatric diagnosed cases

**Table 1:** General characteristics comparison according cohorts

Baseline characteristic	Cohort A *N* = 45 (%)(Dx ≤1994)	Cohort B *N* = 53 (%)(Dx ≥ 1995)	Total (*n* = 98) N (%)	Diff.
Gender M/F (%)	27/18 (60/40)	31/22 (58.5/41.5)	58/40 (59.2/48.8)	NS
Age at Dx (years)	9.0 (05–16.5)	5.7 (0.2–15.9)	7.2 (0.2–16.5)	*p* < 0.001
Patient/sibling (%)	39/6 (86.7/13.3)	47/6 (88.7/11.3)	86/12 (87.7/12.3)	NS
GD1/GD3	40/5 (88.9%/11.1%)	40/13 (75.5%/24.5%)	80/18 (81.6/18.4)	NS
Pediatric Gaucher Severity Index score				
Mild	22 (53.7)	29 (60.4)	51 (52.0)	NS
Moderate	7 (17.1)	17 (35.4)	24 (24.5)	NS
Severe	12 (29.3)	2 (4.2)	14 (14.3)	*P* = 0.045
Symptoms and signs at diagnosis				
Splenomegaly	45 (100)	47 (88.6)	92 (93.9)	NS
Hepatomegaly	27* (60%)	40 (75.5)	67 (68.4)	*p* = 793
Splenectomy	15 (39.5%)	4 (7.8)	19 (19.4)	*p* < 0.001
Anemia or thrombocytopenia	30 (83.3%)	30 (66.7)	60 (61.2)	*p* = 0.047
Mean Hb (range) g/dL	11.4 (8.0–14.7)	11.3 (6.9–13.8)	11.4 (6.9–14.7)	NS
Mean Platelets count (range)	89.3 (21–200) × 10^9^/L	128.3 (44–363) × 10^9^/L	112.9 (21–363)	*p* < 0.001
Other characteristics & biomarkers				
Bone symptomatology	21 (50) (3missing)	12 (26.7) (8 missing)	33 (37.9, 11 missing)	*p* < 0.001
Bone pain (only)	5 (11.9)	3 (6.7)	8 (9.2)	NS
Vascular bone complication	16 (38.1%)	9 (20.0)	25 (25.5)	*p* = 0.025
Chitotriosidase	17,277 (1,123–65,497)	11,038 (370–38,882)	12,437 (370–65497)	NS
CCL18/PARC	271 (151–552)	1,273 (105–3763)	1,023 (105–3,763)	NS
Genotypes GD1				
N370S/N370S	1 (2.2)	1 (1.9)	2 (2.0)	NS
N370S/L444P	13 (28.9)	14 (26.4)	27 (27.5)	NS
N370S/Other	25 (55.6)	22 (41.5)	47 (47.9)	NS
Other/Other	1 (2.2)	3 (6.7)	4 (4.08)	
Genotypes GD 3				
L444P/L444P	0 (0)	7 (13.2)	7 (7.1)	-
L444P/Other	3 (6.7)	2 (3.8)	5 (5.1)	NS
Other/Other	2 (4.4)	4 (7.5)	6 (6.12)	NS

*Dx* diagnosis, *pts* patients

### Genotyping & GD subtypes

**Fig. 2:**
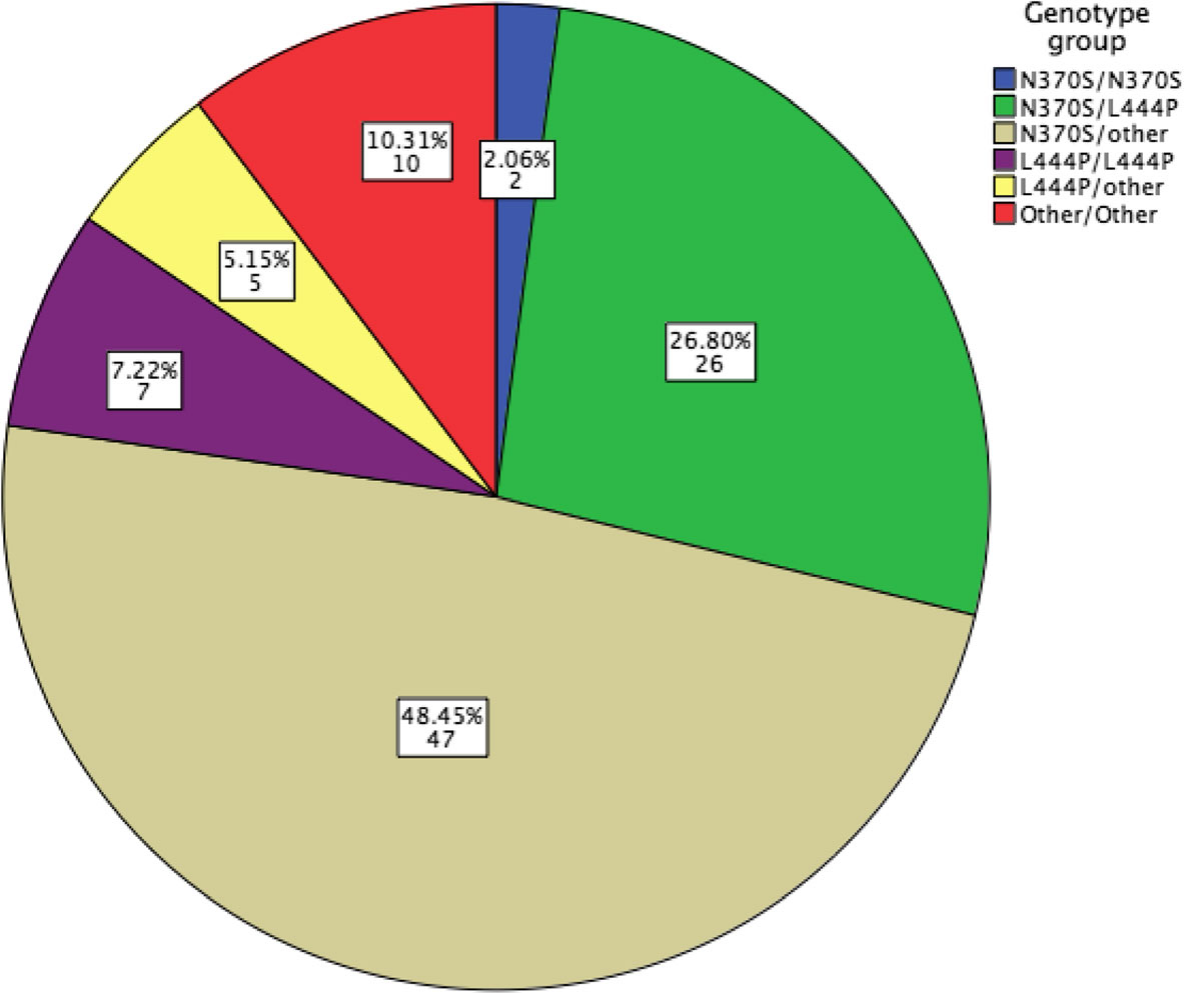
Genotype Distribution

In mutational analysis N370S compound heterozygosis (73, 74.5%) was the most frequent genotype, of which N370S/L444P was the most common (26, 26.5%). Only 2 (2.0%) patients were homozygous for N370S. Regarding the GD3 patients, homozygosity for L444P was the most frequent genotype (7/18, 41.0%) See Fig. 2.

In general, majority of patients (70.4%, 69 cases) carried at least one severe mutation (L444P, D409H, RecNi, 84GG, W(−4X), T391P, del55pb, G202R, G195W, P182L, E326K and others). In 20.4% (20) the severity of the mutation was unknown and the remainder were mild (N370S, G377S), (data not shown) [12].

### Baseline evaluation

During diagnosis or follow-up process 17 patients were splenectomized, three underwent a liver biopsy. There was an association (*p* < 0.001) between the diagnosis before 1994 and splenectomy (14/17 cases), the last three spleen removals occurs in 1998–2001 during the diagnostic process.

According to the PGS3, in GD1 patients (*n* = 80), more than half had mild disease at baseline (41, 56.3%), 19 (26.0%) had moderate and 13 (17.8%) had severe presentations, there were incomplete data in seven cases. When the PGS3 was applied to GD3 patients 10 (62.3%) was classified as mild, five (31.3%) as moderate and 1 (6.3%) as severe. “There were more severe patients in the group diagnosed before 1994 (*p* = 0.045).

### Visceral involvement

Splenomegaly affected all patients; the mean for cohort A was 9.8 (2–23) cm below costal rib (BCR) and for cohort B 7.7 (3–19) cm BCR. Hepatomegaly was more prevalent in cohort A.

### Hematologic involvement

Table 1 details the hematological parameters at baseline and compares the values in the two cohorts. In cohort A, 86.7% (30, missing data = 9) of patients at diagnosis showed anemia and/or thrombocytopenia, with a mean Hb value of 9.1 (8.0–10.5) g/dL and platelet count of 80.9 (21–112) ×10^9^/L, cohort B showed 66.7% (30 cases, 6 missing data) with a mean Hb concentration of 9.7 (6.9–10.9) g/dL and platelets count of 89.3 (44–117) ×10^9^/L. The presence of thrombocytopenia was higher in cohort A (*p* < 0.001) and had lower values (*p* = 0.02) (89.3 vs 128 × 10^9^/L).

### Bone disease

There were clear differences between both cohorts (*p* < 0.001), with more severe disease presentation in cohort A. This was associated with a mean older age at diagnosis for cohort A, 9.0 (0.5–16.5) years, and for cohort B, 5.7 (0.2–15.9) years (*p* < 0.001), an undoubted improvement after therapy availability. In the A cohort, a total of 21 (50%) patients reported bone pain before diagnosis, 16 had reported symptoms that resembled a bone crisis and two patients had suffered vertebral fractures. In the cohort B only 12 (26.7%) reported bone pain at diagnosis and seven reported a bone crisis or/and AVN (4). Two L444P homozygous patients developed Norbottnian-like thoracic deformations before 10 years of age and this was reported during the diagnosis process. There was no correlation between the presentation of bone symptomatology and early diagnosis, however the hazard ratio (HR) to manifest a bone complications was 1.7 times higher in cohort A compared to Cohort B with statistically significant (*p* = 0.025) differences between them.

### Osteopenia

In 13 patients form cohort A osteopenia was assessed and found 5 (38.5%) patients at diagnosis and in cohort B 6/16 (37.5%) assessed patients had osteopenia at diagnosis, there were no differences between the two cohorts. Probably the incidence of osteopenia is under diagnosed due to un-availability of densitometry in all the centers.

### Biomarkers

All the patients showed abnormal values of chitotriosidase, except one with homozygous 24 pb duplication in the *CHIT1* gene with 0 activity; 11 patients were heterozygous for 24 pb duplication in *CHIT1* gene. The mean values for chitotriosidase showed a wide distribution ranging from 1,123 to 65,497 nmol/mgprot.h. For the cytokine CCL18/PARK all the assessed patients showed high abnormal values ranging from 151 to 3,763 ng/mL. The observed differences between cohorts about the biomarkers values were no statically significant.

### Growth assessment

Eight patients (5 from cohort B) were below the ten percentile for height and gender at diagnosis. There were no significant differences regarding the availability of data to calculate the expected height according parents heights, and there were scanty data from the cohort A patients to establish a proper correlation. From the cohort B, all patients achieved a normal weight and improve the percentile for height to normal patterns during therapy.

### GD1 patients (*n* = 80)

Each cohort included 40 GD1 patients with only one N370S homozygous patient in each one and a similar frequency of N370S/L444P patients (Cohort A 13, 32.45% patients, Cohort B 14, 35.0% patients), the most common genotype in our population. Based on mutation analysis performed by our group few years ago [11, 12], the majority of patients (33 and 25 cases in cohort A and B respectively) carried an allele with mutations categorized as severe, i.e. 84GG, del55pb and other null mutations, combined mutations, G195W, G202R, T134P and others (data not published in this article). Regarding the initial symptoms, bone pain was the chief complaint in 20 (50%) of GD1 patients of cohort A while in Cohort B, spleen enlargement was the most common sign and only 12 (30%) GD1 patients reported bone pain in cohort B as was previously described.

### GD3 patients (*n* = 18)

Five patients from cohort A were GD3, none of them L444P homozygous, but carrying L444P (2), D409H (2), G377S (1), G195W (1) or the recombinant mutation E326K + N188S (2) with other mutations. In cohort B, 13 patients were GD3, 7 homozygous L444P, 1 L444P with another mutation and 5 with other mutations: homozygous D409H (1), heterozygous D409H (3), RecNi (2), homozygous P266L, heterozygous R463C (1) or G377S (1).

The initial manifestation of GD3 phenotype were strabismus/oculomotor apraxia in 11, psychomotor retardation in 3, myoclonic epilepsy in 3 and thorax in bunker was present in 2 L444P homozygous patients (one of them with saccadic eyes movement).

From the patients whom access to therapy after childhood, in cohort A (31 cases).

### ERT access

The median follow-up (until last contact or 18 years old) was 8.8 (0.3–17.5) years. During this time 12 patients (26.7%) of cohort A and 42 (79.2%) from cohort B initiated ERT during childhood, (*p* < 0.001), globally 55.1% (54) of cases. The mean global time from diagnosis to ERT for cohort A was: 5.2 (0.1–11.9) years, and for cohort B: 1.6 (0.1–11.2) years (*p* = 0.001). The mean age at ERT for cohort A was 12.2 (2.9–17.1) years and for cohort B: 6.6 (0.2–16.0) years, (*p* = 0.001).

From the patients whom access to therapy after childhood, in cohort A (31 cases, missing data 2 cases), the mean age was 31.1 (18.4–45.9) years and for cohort B there was only one case (missing data, 11 cases) at 18.1 years.

## Discussion

Besides Ashkenazy’s Jewish people, GD is a panethnic and rare disease with a prevalence around 1/70,000–100,000 population [6]. In Spain, the Spanish Registry of Gaucher Disease, has reported 386 patients since the establishment of the registry in 1993 for an updated prevalence of ~ 1:117,000 habitants (www.feeteg.org, updated in May 2016).

At the time the diagnostic process was started by pediatricians, family medicine or other specialists, the main manifestations included alterations in blood counts and spleen enlargement; this generally required a hematological consultation, that led to the diagnosis in approximately 75% of GD patients [31, 32]. In our experience the clinical suspicion based on the presence of organomegaly, anaemia, thrombocytopenia and bone pain had been sufficient to identify the majority of patients, thus avoiding bone marrow aspiration/biopsy.

Around 49% of the cases (reported from the international GD registry, ICCG) are diagnosed before 10 years of age and 66% before 20 years old [15]. Probably an early diagnosis suggests a more aggressive behavior of the disease; in our experience, thanks to the efforts of the FEETEG collaborators, only 34.0% of children with GD were diagnosed after 10 years, and 38.1% were diagnosed before 5 years, an important improvement compared to the international experience. When these data are analyzed regarding the availability or not of therapy, after 1994, 75% of diagnosed patients were bellow 10 years of age and 53.8% bellow 5 years of age, a clear improvement compared to the international experience. However this sub-analysis has not been made with the ICGG Registry data so cannot be compared.

The availability of enzymatic replacement therapy has changed the landscape of Gaucher disease pediatric patients; the starting point was the worldwide introduction in 1991 of Ceredase (Genzyme, Cambridge, USA) and its replacement by the recombinant form imiglucerase (Cerezyme, Genzyme, Cambrigde, USA) in 1994. It took more than 15 years to add two new enzymes to the therapeutic arsenal, velaglucerase alfa (VPRIV, Shire, Cambrigde, USA), identical to the human enzyme, approved in 2010 and taliglucerase alfa (Protalix, Carmel, Israel) the first plant-based enzyme form approved in 2012. The oral substrate reduction therapy is not yet an option for pediatric patients. Enzyme replacement therapy (ERT) is a non-curative and expensive therapy, is effective in alleviating the visceral manifestations, decreasing bone marrow burden(BMB)/infiltration and preventing bone crisis and bone lesions [33, 34].

Despite the availability of ERT, the key diagnosis features of type 1 Gaucher Disease (GD1) are still the hematologic alterations like low hemoglobin concentration, low platelet count, bone pain/bone crisis, hepato- and splenomegaly [32]. Splenomegaly was almost universal in our pediatric patients presentation, 66.7–83.3% presented with hematologic alterations and 26.7–50% with bone manifestations. This is consistent with other reports including the ICGG Registry [13, 30, 33] and a call for increased awareness in the hematologic field to identified these patients.

The goals of therapy by Pastores et al. [22] are based on the improvements achieved by at least 80% of the patients treated with enzymatic replacement therapy and this needs to be optimized according the different presentation of the patients. Working with rare disease is a continual challenge, and more in a pediatric setting, the initial problem is, in our experience, to spread widely the knowledge to physicians to recognize the disease and to carry out an extensive baseline assessment to identified the patients in need of therapy and thus avoid complications [35]. Pediatric patients usually reflect more physiologic alterations, with high risk for bone disease and chronic growth failure [15]. The current recommendations for start ERT on children [22], are based in the presence of symptomatology, growth tendency, bone, visceral and hematological alterations. In patients with severe genotypes such L444P and D409H or other genotypes that suggest the diagnosis of a GD3 case, is recommended to initiate therapy as soon as possible. For asymptomatic patients diagnosed bellow 20 years old, it is recommended to start ERT if they are sibling of a patient who requires therapy.

In this respect, it should not be forgotten that the prevalence of genotypes varies among the different populations; in Ashkenazi Jews, N370S is the most frequent mutation [36] and more than the 40% of those pediatric patients are homozygous for N370S mutation [37], a rare feature in our population (only 2 cases, 2.0%). In our cohort there was a predominance of L444P compound heterozygotes and other severe mutations, in line with the more aggressive phenotypes. The data from the international Gaucher Registry also show a lower frequency of N370S/L444P genotype than in our population (14.8%) and a higher presence of N370S in homozygosity (7.9%) [15]. This shows the necessity to establish local recommendations based on population characteristics.

In respect to bone crisis, data from the IGGC registry report only 17% of patients with bone crisis [15], from the UK registry study 26% GD1 patients reported bone pain [17], but in our cohort, bone alterations were present in 37.9% (33) of cases, with a clear predominance (50%, 21 cases) in the cohort diagnosed before 1994 than the patients diagnosed after ERT became available. This is a clear feature of a more severe presentation in our country.

## Conclusions

According to the data including in the Spanish Gaucher Disease Registry the mean age at diagnosis in childhood has changed from 9.0 to 5.7 years in the last 21 years.

In the Spanish population there is greater tendency to severe phenotypes and genotypes in pediatric population compared with ICGGR, probably related to the different genotype spectrum. In our cohort, excluding the siblings, the patients were symptomatic and following the current recommendation in need of therapy. The indications for therapy based on the presence of severe mutations have to be considered according the local spectrum of mutations and behavior of patients. The presence of infrequent mutations like R463C, G377S, P266L and recombinant alleles need to be considered among the indicators for therapy.

Related to bone disease, the higher prevalence of bone complications in children with delayed start of therapy is remarkable, in our study 1.7 times higher in children diagnosed before 1994.

Comparing the patients diagnosed before or after the availability of ERT this aspect has improved. Physicians need to be careful and recognize patients with less severe manifestation in order to initiate therapy early and avoid the disease complications. These changes are related with the increased awareness of physicians and the efforts made to diagnose patients after ERT became available.

## Abbreviations

AVN: Avascular necrosis
BCR: Below costal ribs
BMB: Bone marrow burden
BUA: Broadband ultrasound attenuation
CCL18/PARC: Chemokine-C-motif ligand 18/Pulmonary-activation-regulated chemokine
CHIT: Chitotriosidase
cohort A: ≤ 1994
cohort B: ≥1995
DXA: Dual X-ray absorptiometry
ERT: Enzymatic replacement therapy
EWGGD: European Working Group on Gaucher Disease
FEETEG: Fundacion Española para el Estudio y Terapeutica de la Enfermedad de Gaucher
GBA: Glucocerebrosidase
GD: Gaucher disease
GD1: Type 1 Gaucher disease
GD3: Type 3 Gaucher disease
Hb: Hemoglobin
HR: Hazard ratio
ICGGR: International Collaborative Gaucher Group Registry
PGS3: Pediatric Gaucher severity scoring system
SpRGD: Spanish Registry of Gaucher Disease
WHO: World Health Organization
